# Supplementary material for: Nuclear receptor NR2F6 inhibition potentiates responses to PD-L1/PD-1 cancer immune checkpoint blockade
Source: Nat Commun. 2018 Apr 18;9:1538. doi: 10.1038/s41467-018-04004-2 (PMC5906604; doi:10.1038/s41467-018-04004-2)
Supplement: Supplementary file 1 — Supplementary Information [file 41467_2018_4004_MOESM1_ESM.pdf]

Supplementary Information PDF

Nuclear Receptor NR2F6 Inhibition Potentiates Responses to PD-L1/PD-1 Cancer Immune Checkpoint Blockade

Victoria Klepsch et al.

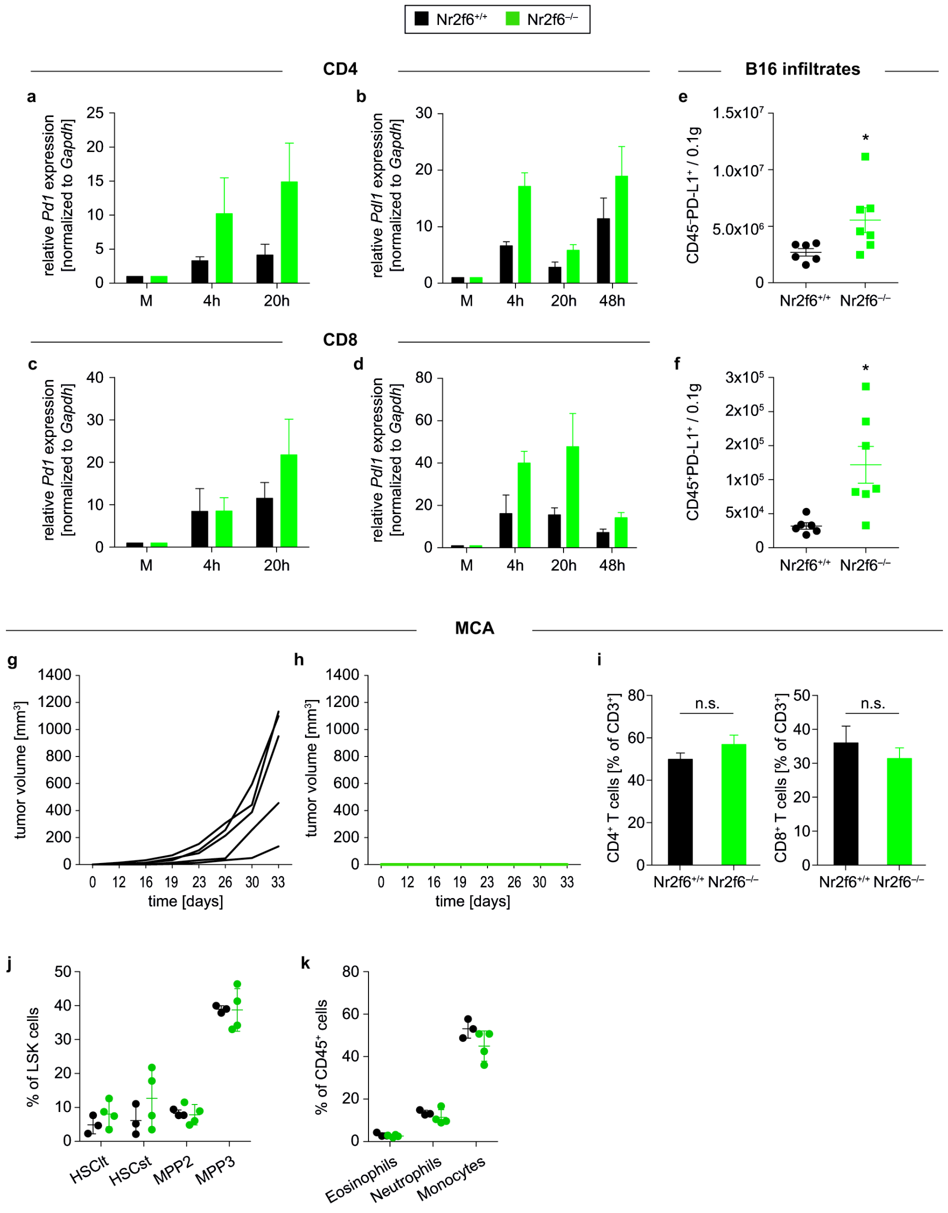

Supplementary Figure 1: NR2F6 inhibition led to an elevated *Pd1* and *Pdl1* expression in $CD4^+$ and $CD8^+$ T cells *ex vivo*. *In vitro* qRT-PCR analysis of *Pd1* mRNA (A, p=0.045, ANOVA), *Pdl1 mRNA* (B, p=0.012, ANOVA) in wild-type and *Nr2f6*-deficient $CD4^+$ T cells as well as in $CD8^+$ T cells (C n.s. D p=0.045, ANOVA) during activation with anti-CD3 mAb (5 µg) and anti-CD28 mAb (1 µg) at the indicated time points (n=4). (E) Increased absolute cell numbers of B16-OVA tumor-infiltrating $CD45^-PD\text{-}L1^+$ non-immune cells (p=0.041, unpaired t-test) and (F) $CD45^+PD\text{-}L1^+$ immune cells (p=0.011, unpaired t-test) per 0.1 g of tumor tissue harvested on d14 after tumor inoculation (*Nr2f6*$^{+/+}$ n=6, *Nr2f6*$^{-/-}$ n=7). Single tumor growth curves of *Nr2f6*$^{+/+}$ (G) and *Nr2f6*$^{-/-}$ (H) mice injected with a 3-MCA generated wild-type cell line (respective to Fig.1D). (I) No difference of CD4 and CD8 distribution in *Nr2f6*$^{-/-}$ mice compared to controls in the $CD3^+$ pool of TILs from MCA-induced tumors were analysed at the endpoint (n=5). (J) Frequency of myeloid stem cell and progenitor populations as HSC^{LT}, HSC^{ST}, MMP2 and MMP3 in the BM of *Nr2f6*$^{+/+}$ or *Nr2f6*$^{-/-}$ B16-tumor bearing mice on day 14. (K) Frequency of myeloid cell populations such as eosinophils, neutrophils or monocytes in the BM of *Nr2f6*$^{+/+}$ or *Nr2f6*$^{-/-}$ tumor bearing mice on day 14 (n=4). Results shown are derived from at least two independent experiments. Error bars represent the mean ± SEM.

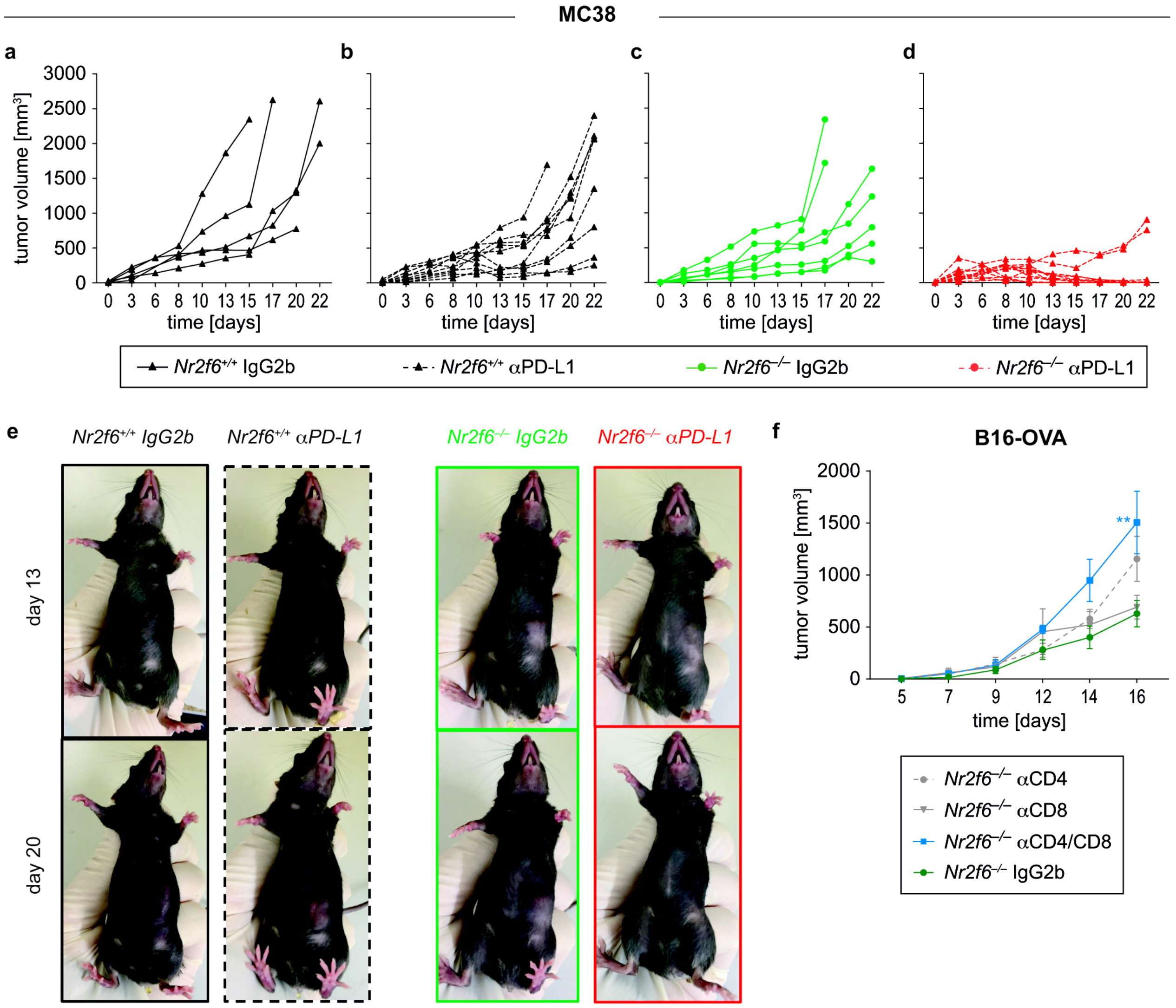

Supplementary Figure 2: (A-D) Single tumor growth curves in experiments shown in Fig.2C and D of (A) *Nr2f6*$^{+/+}$ mice receiving isotype control (Rat IgG2b), (B) *Nr2f6*$^{+/+}$ mice receiving antibody against PD-L1, (C) *Nr2f6*$^{-/-}$ mice receiving isotype control (Rat IgG2b) and (D) *Nr2f6*$^{-/-}$ mice receiving an antibody against PD-L1. (E) Representative pictures of tumor-bearing mice on d13 and d20 after MC38 tumor injection of the four already described groups. (F) Tumor cell growth kinetics in *Nr2f6*$^{-/-}$ mice treated with CD8^{+} (grey triangle, p=0.9524) CD4^{+} (grey circle, p=0.051, ANOVA), CD4^{+}/CD8^{+} combination (blue, p=0.0017, ANOVA) neutralizing antibodies or rat IgG2b (green) and injected with 1x10^{5} B16-OVA melanoma cells (n=4). Results shown are derived from at least two independent experiments. Error bars represent the mean ± SEM.

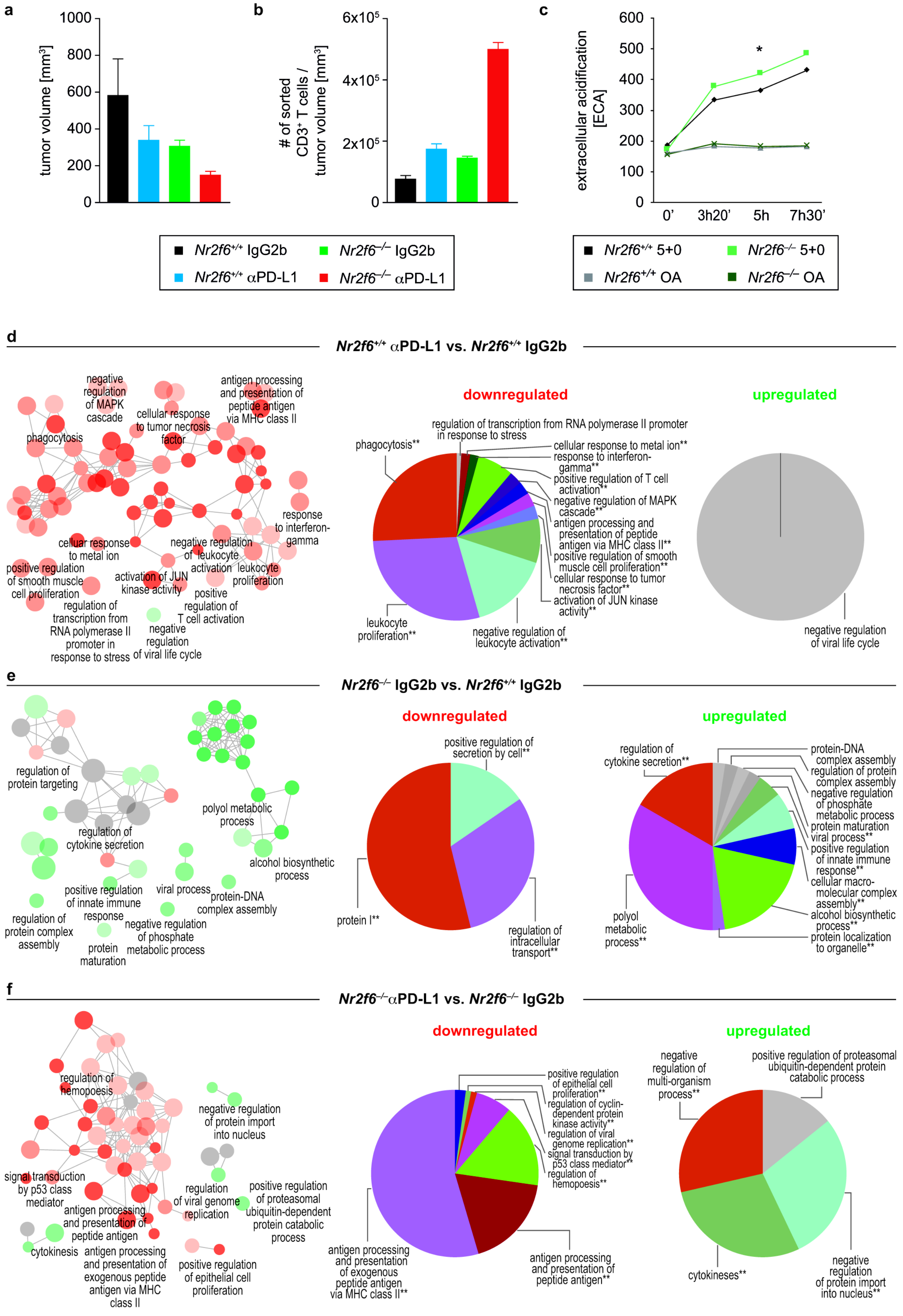

Supplementary Figure 3

Supplementary Figure 3: (A) Tumor volume (mm^3) of $5x10^5$ B16-OVA melanoma cells injected s.c. in *Nr2f6*$^{+/+}$ or *Nr2f6*$^{-/-}$ mice treated with IgG2b (n=4) or αPD-L1 (n=8) with significant differences between *Nr2f6*-deficient mice treated with IgG2b or αPD-L1 (p=0.013, unpaired t-test) and αPD-L1-treated *Nr2f6*$^{+/+}$ and *Nr2f6*$^{-/-}$ mice (p=0.033, unpaired t-test). (B) As well as significant differences of absolute sorted $CD3^+$ cell numbers in *Nr2f6*$^{-/-}$ mice treated with αPD-L1 (compared to *Nr2f6*$^{-/-}$ IgG2b p<0.0001 or *Nr2f6*$^{+/+}$ IgG2b p<0.0001, unpaired t-test), additionally αPD-L1 treatment in wild-type mice also revealed significantly increased $CD3^+$ cell numbers (p=0.003, unpaired t-test) compared to the wild-type IgG2b group; likewise, monotherapy of *Nr2f6* loss showed elevated infiltration of $CD3^+$ T cells (p=0.024, unpaired t-test). (C) MACS sorted $CD3^+$ T cells of wild-type (black, n=5) and *Nr2f6*$^{-/-}$ (green, n=5) mice were stimulated with anti-CD3 / anti-CD28 for three days and showed a significant higher extracellular acidification (p=0.031, ANOVA) using the MitoXpress® – pH Xtra™ Glycolysis Assay Kit from Luxcel (OA = Oxamic acid, negative control). (D-F) Additional data of RNAseq experiments showing ClueGO gene ontology analysis of significantly differentially expressed genes in the control groups of sorted $CD3^+$ TILs of B16-OVA tumors (D). Results shown are derived from at least two independent experiments. Error bars represent the mean ± SEM.

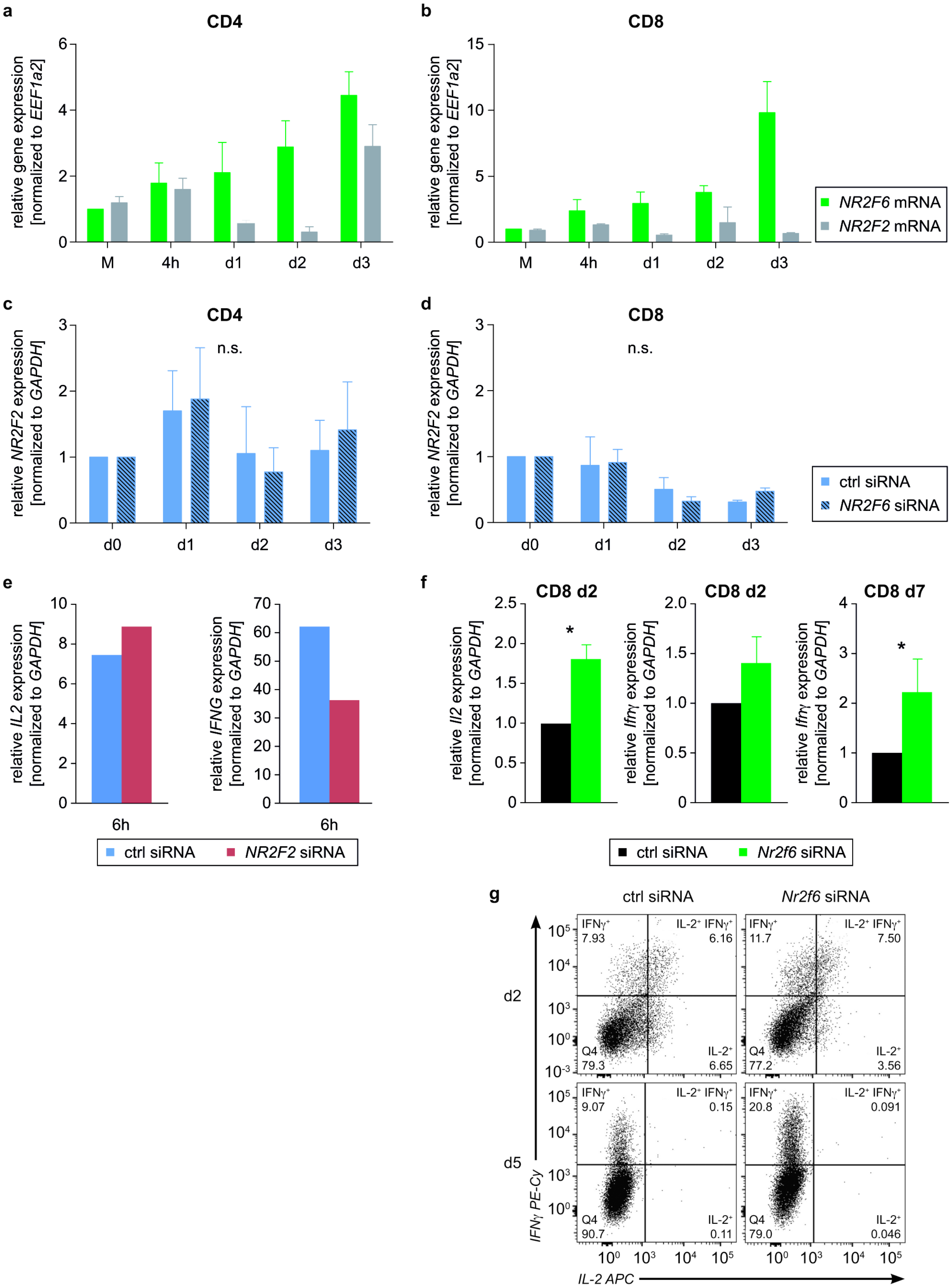

Supplementary Figure 5

Supplementary Figure 5: Human *NR2F6* mRNA (green) is upregulated upon stimulation of $CD4^+$ (A) or $CD8^+$ (B) isolated T cells from human PBMCs in a time-dependent manner (n=3; CD4 p=0.0084, CD8 p=0.001, ANOVA). The two other family members of the COUP-TF nuclear receptor family are not significantly upregulated upon stimulation of T cells (A,B shows mRNA expression of *NR2F2* (blue) and *NR2F1* is not detectable in human T cells). Upon gene silencing of *NR2F6* via siRNA transfection, the expression of *NR2F2* is not upregulated to rescue the loss of *NR2F6* compared to ctrl siRNA in $CD4^+$ (C) and $CD8^+$ (D) T cells stimulated over 3 days (n=3). (E) siRNA-mediated knock-down of the family member *NR2F2* in T cells does not alter their function upon stimulation measured by *IL2* and *IFNG* mRNA expression compared to *control* siRNA, indicating a non-relevant role of *NR2F2* in human T cells. (F) Mouse $CD8^+$ T cells were nucleofected with *Nr2f6* siRNA or *control* siRNA as indicated (n=3). Cytokine expression levels were analyzed by qRT-PCR on day 2 after transfection following 4 hours of PDBu (0.05 µg/ml)/ionomycin (0.5 µg/ml) stimulation or on day 7 after transfection during activation with anti-CD3 mAb (5µg) and anti-CD28 mAb (1 µg) and *Nr2f6* siRNA treated cells showed significant elevated levels of *Il2* (p=0.02, unpaired t-test) on day 2 and *Ifnγ* on day 7 (p=0.02, unpaired t-test) and a trend for *Ifng* on day 2. (G) Representative dot plots show elevated levels of IFNγ on day 2 (PDBu/ionomycin) and day 5 (anti-CD3/CD28) after transfection in $CD8^+$ cells transfected with *Nr2f6* siRNA in comparison to *control* siRNA. Scale bars = 200 µm (a-d, upper panels) and 100 µm (a-d, lower panels). Results shown are derived from at least two independent experiments. Error bars represent the mean ± SEM.

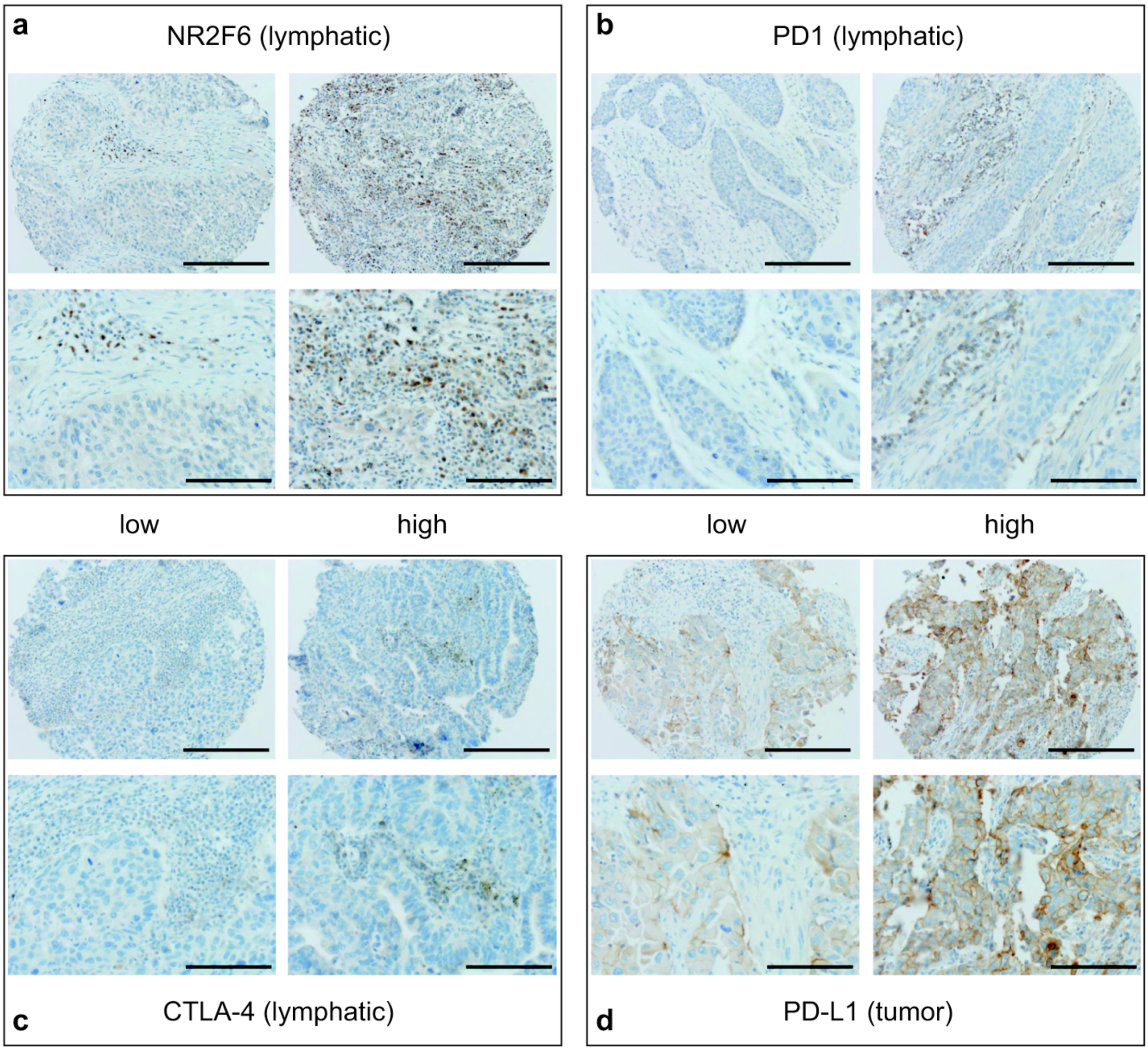

Supplementary Figure 6: Representative IHC images for all antigens reported in the multiplex analysis of Fig.6E are shown. Scale bars = 200 µm (a-d, upper panels) and 100 µm (a-d, lower panels).

Uncropped immunoblots used in Fig. 5i

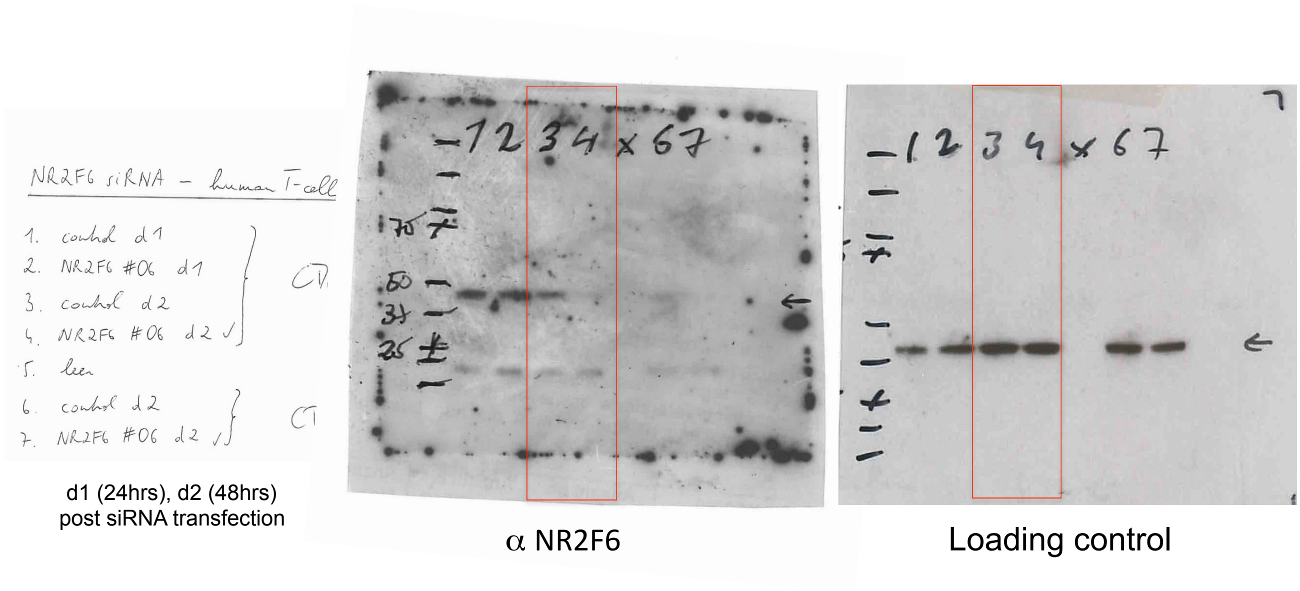

Supplementary Figure 7: Source data of western blots in Fig. 5i

Supplementary Table 1: *Nr2f6*$^{-/-}$ IgG2b vs *Nr2f6*$^{+/+}$ IgG2b

p---value	pathway	associated genes
0,0003	Cell cycle	P+g1; Mad2l1; Ccna2; Mcm2
0,0008	Oxidative phosphorylation	Ndufv3; Gzmb; Atp5f1
0,0040	APC/C:Cdc20 mediated degradation of mitotic proteins	P+g1; Mad2l1
0,0053	Regulation of DNA replication	Ccna2; Mcm2
0,0097	Separation of Sister Chromatids	P+g1; Mad2l1; Cenpa

p---value	pathway	associated genes
0,0005	Growth hormone receptor signaling	Jak2; Cish
0,0006	Steroid biosynthesis	Lipa; Ebp
0,0009	Morphine addiction	Pde7a; Gnb2; Pde3b
0,0041	Jak---STAT signaling pathway	Jak2; Cish; Il4ra
0,0053	Purine metabolism	Pde7a; Pola1; Pde3b
0,0089	Kit Receptor Signaling Pathway	Jak2; Cish
0,0054	Endometrial cancer	Tcf7; Axin1
0,0061	Basal cell carcinoma	Tcf7; Axin1
0,0081	Colorectal cancer	Tcf7; Axin1

Supplementary Table 2: *Nr2f6*$^{-/-}$ PD-L1 vs *Nr2f6*$^{+/+}$ PD-L1

p---value	pathway	associated genes
0,0000	Osteoclast differentiation	Nfatc1; Nfatc2; Fhl2; Stat1; Junb; Socs3; Cybb; Fosl2; Ppp3cc
0,0001	Natural killer cell mediated cytotoxicity	Nfatc1; Nfatc2; Klrc2; Cd48; Klra3; Ppp3cc; Klrd1
0,0004	Interferon gamma signaling	Socs3; Stat1; Camk2b
0,0006	T cell receptor signaling pathway	Nfatc1; Nfatc2; Cd28; Il10; Prkcq; Ppp3cc
0,0009	Immune System	Nfatc1; Nfatc2; Adcy7; Stat1; Socs3; Hectd3; H2---DMa; Dapp1; Camk2b; Rps6kb2; Cybb; Ripk3; Cd28; Tnfaip3; Prkacb; Prkcq; Klrc2; Zbp1; Klrd1
0,0016	Graa---versus---host disease	H2---DMa; Cd28; Klra3; Klrd1
0,0025	Innate Immune System	Nfatc1; Nfatc2; Cd28; Klrd1; Adcy7; Zbp1; Tnfaip3; Rps6kb2; Ripk3; Klrc2; Prkacb; Prkcq
0,0029	Wnt signaling pathway	Nfatc1; Nfatc2; Siah1b; Camk2b; Prkacb; Ppp3cc
0,0033	DAP12 signaling	Cd28; Klrd1; Adcy7; Rps6kb2; Klrc2; Prkacb
0,0042	Interleukin---6 signaling	Socs3; Stat1
0,0042	Type II interferon signaling (IFNG)	Cybb; Socs3; Stat1
0,0045	TNF signaling pathway	Ripk3; Junb; Tnfaip3; Socs3; Ccl5
0,0049	Signaling by PDGF	Cd28; Stat1; Rps6kb2; Adcy7; Spp1; Prkacb
0,0051	Regulation of IFNG signaling	Socs3; Stat1
0,0059	B cell receptor signaling pathway	Nfatc1; Nfatc2; Dapp1; Ppp3cc
0,0077	Intestinal immune network for IgA production	Il10; H2---DMa; Cd28
0,0094	PKA activation	Adcy7; Prkacb

p---value	pathway	associated genes
0,0040	Nucleosome assembly	Cenpo; Ruvbl1; Cenpk
0,0040	TAK1 activates NFkB by phosphorylation and activation of IKKs complex	Neb2; Ripk2; Hmgb1
0,0046	RNA transport --- Mus musculus (mouse)	Nup54; Eef1a1; Rpp30; Gemin6; Gemin7; Alyref2; Eif3a
0,0065	Activation, myristolyation of BID and translocation to mitochondria	Gzmf; Gzmc
0,0065	Calcitonin---like ligand receptors	Ramp1; Ramp3
0,0077	TRAF6 Mediated Induction of proinflammatory cytokines	Map2k2; Neb2; Ripk2; Hmgb1
0,0079	Recruitment of NuMA to mitotic centrosomes	Tubgcp2; Tubgcp6
0,0085	Signal transduction by L1	Map2k2; Itgb3; Ap2a1

Supplementary Table 3: *Nr2f6*$^{+/+}$ PD-L1 vs *Nr2f6*$^{+/+}$ IgG2b

p---value	pathway	associated genes
0,0002	Chromosome Maintenance	Cenpo; Ruvbl1; Rfc2; Hist1h3c; Pole; Atr
0,0002	Ribosome --- Mus musculus (mouse)	Rpl22l1; Mrpl11; Mrpl22; Rpl36; Rpl37; Mrps18a; Mrpl2
0,0004	glycolysis V (Pyrococcus)	Aldoc; Pgam1; Pkm
0,0008	Oxidative phosphorylation	Ndufs6; Nduh2; Ndufa8; Atp5l
0,0009	Fanconi Anemia pathway	Fancf; Atr; Fanci
0,0022	DNA Repair	Fancf; Pole; Atr; Fanci; Rfc2
0,0023	Glycolysis	Aldoc; Pkm; Pgam1
0,0025	Cell Cycle	Cenpo; Ruvbl1; Cdc25a; Nup85; Rfc2; Hist1h3c; Pole; Atr; Cdca8; Anapc7
0,0037	Cell Cycle Checkpoints	Cdc25a; Atr; Anapc7; Rfc2
0,0068	Electron Transport Chain	Nduh2; Ndufa8; Ndufs6; Atp5l
0,0080	Gap---filling DNA repair synthesis and ligation in GG---NER, TC---NER	Pole; Rfc2

Supplementary Table 4: *Nr2f6*$^{+/+}$ PD-L1 vs *Nr2f6*$^{+/+}$ IgG2b

p---value	pathway	associated genes
0,0000	Osteoclast differentiation	Nfatc2; Fcgr4; Socs3; Sirpa; Fos; Tyrobp; Fosb; Junb; Jun; Cybb; Syk; Fosl2; Ppp3cc; Fcgr3
0,0000	Cytokine---cytokine receptor interaction	Il7r; Ccl2; Csf2ra; Ccl6; Il6ra; Il10rb; Ccl8; Ccl9; Ccr7; Tnfrsf21; Cxcl1; Ppbp; Cxcr4; Tnfrsf13b; Ccl7
0,0000	Classical antibody---mediated complement activation	Igkc; C1qc; C1qb; C1qa
0,0000	IL---6 signaling Pathway	Il6ra; Socs3; Fos; Jun; Fgr; Ar; Hck; Lyn; Crebbp
0,0000	Immune System	Ifitm2; Il6ra; Socs3; Fos; Tyrobp; C1qc; Camk2b; Csf2ra; Bcl10; Cd74; Lgmn; Syk; Jun; C1qb; C1qa; H2---Eb1; Rnf41; Il7r; Nfatc2; Nr4a1; Map3k1; H2---Aa; H2---DMb1; Tnfaip3; Lyn; Crebbp; App; Cybb; Unc93b1; Igkc
0,0000	Chemokine signaling pathway	Ccl2; Cxcl1; Cclt9 Ccl6; Ccl7; Ccl8; Cxcr4; Fgr; Ppbp; Ccr7; Hck; Lyn
0,0000	Intestinal immune network for IgA production	H2---Ab1; H2---Aa; H2---DMb1; H2---Eb1; Cxcr4; Tnfrsf13b
0,0001	Creation of C4 and C2 activators	Igkc; C1qc; C1qb; C1qa
0,0001	B cell receptor signaling pathway	Nfatc2; Fos; Syk; Jun; Lyn; Ppp3cc; Bcl10
0,0002	T Cell Receptor Signaling Pathway	Nfatc2; Fos; Syk; Map3k1; Jun; Lax1; Lyn; Bcl10; Crebbp
0,0003	TNF signaling pathway	Ccl2; Cxcl1; Socs3; Fos; Junb; Jun; Tnfaip3; Ak4
0,0003	Innate Immune System	Nfatc2; Map3k1; Lyn; Lgmn; Fos; Syk; Jun; App; Nr4a1; C1qc; C1qb; C1qa; Tyrobp; Tnfaip3; Unc93b1; Igkc; Bcl10; Crebbp
0,0003	MHC class II antigen presentation	Cd74; H2---DMb1; H2---Aa; H2---Eb1; Lgmn
0,0010	Antigen processing and presentation	Cd74; Lgmn; H2---Ab1; H2---Aa; H2---DMb1; H2---Eb1
0,0012	Phagosome	Mrc1; H2---Ab1; H2---Aa; Cybb; H2---DMb1; H2---Eb1; Fcgr4; Fcgr3; Msr1
0,0029	MAPK signaling pathway	Nr4a1; Fos; Map3k1; Jun; Dusp5; Ak4; Ppp3cc; Dusp1
0,0037	IL---5 Signaling Pathway	Syk; Hck; Lyn; Alox5ap; Jun
0,0065	Allograa rejection	H2---Ab1; H2---DMb1; H2---Aa; H2---Eb1
0,0075	Graa---versus---host disease	H2---Ab1; H2---DMb1; H2---Aa; H2---Eb1
0,0079	Natural killer cell mediated cytotoxicity	Nfatc2; Fcgr4; Tyrobp; Fcer1g; Syk; Ppp3cc
0,0080	TGF Beta Signaling Pathway	Jun; Crebbp; Tgif1; Fos
0,0082	Adaptive Immune System	Cd74; Ifitm2; Socs3; Lgmn; Syk; Nr4a1; H2---Aa; Rnf41; Cybb; H2---DMb1; Lyn; H2---Eb1; Igkc; Bcl10
0,0086	Lipoprotein metabolism	Apoe; Pltp; Lpl
0,0086	Oxidative Stress	Junb; Hmox1; Fos
0,0096	Interleukin---6 signaling	Il6ra; Socs3
0,0096	Translocation of ZAP---70 to Immunological synapse	H2---Aa; H2---Eb1
0,0096	Activation of the AP---1 family of transcription factors	Jun; Fos

Supplementary Table 5: *Nr2f6*$^{-/-}$ PD-L1 vs *Nr2f6*$^{-/-}$ IgG2b

p---value	pathway	associated genes
0,0000	Asparagine N---linked glycosylation	Man2a2; Alg5; Edem3; Gfpt1; B4galt3; Alg14; Dpm1; Fut8
0,0001	N---Glycan biosynthesis	Man2a2; Alg5; B4galt3; Alg14; Dpm1; Fut8
0,0001	Post---translational protein modification	Sumf1; Man2a2; Pigm; Edem3; Gfpt1; B4galt3; Alg5; Alg14; Dpm1; Fut8
0,0001	SNARE interactions in vesicular transport	Gosr2; Gosr1; Bnip1; Bet1l; Stx16
0,0003	dolichyl---diphosphooligosaccharide biosynthesis	Pigm; Dpm1; Alg5
0,0011	Reactions specific to the complex N---glycan synthesis pathway	Man2a2; Fut8
0,0015	Synthesis of substrates in N---glycan biosythesis	Gfpt1; Dpm1; Alg5
0,0050	fa+y acid β---oxidation IV (unsaturated, even number)	Decr2; Hadha
0,0053	3---phosphoinositide biosynthesis	Pik3c3; Pi4kb; Pik3ip1
0,0056	Separation of Sister Chromatids	Ube2c; Sgol1; Cdc20; Cdca5; Ckap5; Cenpp
0,0074	Mitotic Metaphase and Anaphase	Ube2c; Sgol1; Cdc20; Cdca5; Ckap5; Cenpp
0,0078	NOD---like receptor signaling pathway --- Mus musculus (mouse)	Mapk14; Pstpip1; Nod1; Ccl5
0,0089	Immune System	Klrc1; Rps6ka1; Pdgh; Asb6; Arih2; Lat2; Amica1; H2---T23; Tec; Ctsd; Cdc20; Ripk1; Mapk14; Nod1; Pstpip1; Lat; Flnb; Pik3c3; Cd200r4; Ptpn1; Ppm1b

Supplementary Table 6: *Nr2f6*$^{-/-}$ PD-L1 vs *Nr2f6*$^{-/-}$ IgG2b

p---value	pathway	associated genes
0,0001	Formyl pepide receptors bind formyl pepides and many other ligands	Anxa1; App; Ccl9; Ccl6
0,0001	Antigen processing and presentation	Cd74; Lgmn; H2---Ab1; H2---Aa; Ifi30; H2---DMb1; H2---Eb1; Ctss
0,0001	Innate Immune System	Lgmn; Ube2n; Tyrobp; C1qc; Tnfaip3; P2rx7; Trim32; Nebia; Jun; C1qb; Itgam; Ctss; Dtx4; Tank; Cdkn1a; Nr4a1; Mnda; Itpr1; App; Unc93b1; Fos; Ly86
0,0001	Osteoclast differentiation	Itgb3; Sirpa; Fos; Tyrobp; Csf1r; Fosb; Nebia; Jun; Fosl2; Fcgr3
0,0001	TGF---beta Receptor Signaling Pathway	Snx2; Fos; Cd44; Fosb; Kpnb1; Jun; Pias2; Skil; Dab2; Cdkn1a; Tgif1
0,0001	Toll---Like Receptors Cascades	Nebia; Lgmn; Fos; App; Ube2n; Jun; Itgam; Unc93b1; Ctss; Ly86
0,0007	Adaptive Immune System	Cd74; Ifitm3; Nebia; Lgmn; Ube2n; Cd81; Trim32; Cdkn1a; Nr4a1; H2---Aa; Ifi30; H2---DMb1; Ctss; H2---Eb1; Wsb1; Itpr1; Mgrn1; Pja2; Klhl9
0,0010	Inte9nal immune network for IgA production	H2---Aa;H2---DMb1;Tnfrsf13b;H2---Eb1;H2---Ab1
0,0013	Trafficking and processing of endosomal TLR	Unc93b1; Ctss; Lgmn
0,0023	Chemokine signaling pathway	Ccl2; Ccl1; Ccl6; Ccl8; Nebia; Tiam1; Ccl9; Ccr7; Cxcl10; Gnai3
0,0032	Cytokine---cytokine receptor interaction	Csf2rb; Il2ra; Ccl1; Ccl6; Ccl8; Ccl9; Csf1r; Ccl2; Tnfrsf9; Ccr7; Cxcl10; Tnfrsf13b
0,0032	Downstream TCR signaling	Nebia;H2---Aa;H2---Eb1;Ube2n
0,0034	Signal regulatory protein (SIRP) family interactions	Tyrobp; Sirpa
0,0038	Toll Like Receptor 4 (TLR4) Cascade	Nebia; Fos; App; Ube2n; Jun; Itgam; Ly86
0,0044	TNF signaling pathway	Ccl2; Casp3; Fos; Nebia; Jun; Tnfaip3; Cxcl10
0,0049	Phagosome	Itgb3; H2---Ab1; H2---Aa; H2---DMb1; Tfrc; Itgam; Ctss; Fcgr3; H2---Eb1
0,0059	Hypertrophy Model	Dusp14; Ifrd1; Nr4a3
0,0087	Downregulation of SMAD2/3:SMAD4 transcriptional activity	Trim33; Skil; Tgif1
0,0089	IL---5 Signaling Pathway	Csf2rb; Nebia; Jun; Itgam; Alox5ap
0,0091	Apoptosis induced DNA fragmentation	Kpnb1; Casp3
0,0098	RIG---I/MDA5 mediated induction of IFN---alpha/beta pathways	App; Nebia; Tank; Tnfaip3
